# Single‐cell analyses reveal suppressive tumor microenvironment of human colorectal cancer

**DOI:** 10.1002/ctm2.422

**Published:** 2021-06-06

**Authors:** Yan Mei, Weiwei Xiao, Hao Hu, Guanming Lu, Lingdan Chen, Zhun Sun, Mengdie Lü, Wenhui Ma, Ting Jiang, YuanHong Gao, LiRen Li, Gong Chen, Zifeng Wang, Hanjie Li, Duojiao Wu, Pinghong Zhou, Qibin Leng, Guangshuai Jia

**Affiliations:** ^1^ Affiliated Cancer Hospital and Institute of Guangzhou Medical University State Key Laboratory of Respiratory Disease Guangzhou China; ^2^ Department of Pathology, Guangdong Provincial People's Hospital Guangdong Academy of Medical Sciences Guangzhou China; ^3^ Department of Radiation Oncology Sun Yat‐sen University Cancer Center Guangzhou China; ^4^ Endoscopy Center Zhongshan Hospital, Fudan University Shanghai China; ^5^ Department of Breast and Thyroid Surgery Affiliated hospital of Youjiang Medical University for Nationalities Baise Guangxi China; ^6^ Department of General Surgery, Nanfang Hospital Southern Medical University Guangzhou China; ^7^ State Key Laboratory of Oncology in South China, Collaborative Innovation Center for Cancer Medicine Sun Yat‐Sen University Cancer Center Guangzhou China; ^8^ Department of Colorectal Surgery Sun Yat‐sen University Cancer Center Guangzhou China; ^9^ Center for Synthetic Immunology, Shenzhen Institute of Synthetic Biology, Shenzhen Institutes of Advanced Technology Chinese Academy of Sciences Shenzhen China; ^10^ Institute of Clinical Science, Zhongshan Hospital, Shanghai Institute of Clinical Bioinformatics Fudan University Shanghai China

**Keywords:** human colorectal cancer, scATAC‐seq, scRNA‐seq, tumor immune microenvironment

## Abstract

Profiling heterologous cell types within tumors is essential to decipher tumor microenvironment that shapes tumor progress and determines the outcome of therapeutic response. Here, we comprehensively characterized transcriptomes of 34,037 single cells obtained from 12 treatment‐naïve patients with colorectal cancer. Our comprehensive evaluation revealed attenuated B‐cell antigen presentation, distinct regulatory T‐cell clusters with different origin and novel polyfunctional tumor associated macrophages associated with CRC. Moreover, we identified expanded *XCL1^+^* T‐cell clusters associated with tumor mutational burden high status. We further explored the underlying molecular mechanisms by profiling epigenetic landscape and inferring transcription factor motifs using single‐cell ATAC‐seq. Our dataset and analysis approaches herein provide a rich resource for further study of the impact of immune cells and translational research for human colorectal cancer.

## Graphical abstract

1) Single‐cell profiling of clinical samples of 12 human colorectal cancer patients.

2) Tumor mutational burden states of human CRC contribute to distinct immune profile patterns.

3) Single‐cell analysis in human CRC identified phenotypic and functional diversity of tumor‐associated macrophages and T cells.

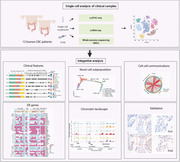

## 1 INTRODUCTION

Tumor microenvironment (TME) influences both tumor progress and response to immunotherapy.^1^, ^2^ Single‐cell RNA sequencing (scRNA‐seq) provides unprecedented detailed characterization of transcriptomes of cell diversity and heterogeneity thereby allowing comprehensive assessment of the complexity of tumor microenvironment.^3^ Numerous single‐cell RNA‐seq studies have been carried out in human colorectal cancer (CRC) focusing on organoid, non‐immune cells, T cells, myeloid cells and multimodal omics analysis,^4^, ^5^, ^6^, ^7^, ^8^, ^9^ however, the TME heterogeneity and the crosstalk with non‐immune cells in human CRC remains to be elucidated.

CRC occurs globally with high mortality as the third most common cancer.^10^ Accumulated genetic alterations in oncogenes and tumor suppressor genes (e.g., *APC, KRAS, PIK3CA*, etc.) drive CRC tumorigenesis.^11^ In addition, dysfunction of DNA mismatch repair genes (MMR) that lead to instability of the microsatellite pathway (MSI) and genetic hypermutability is also associated with sporadic CRC tumorigenesis.^11^ As a result of the complexity of CRC tumorigenesis and clinical status, a combination of surgery, chemotherapy, radiation as well as immunotherapy has been applied to treat CRC patients. Actually, MSI is a good prognostic marker for CRC patients who benefit from targeting immune checkpoint by PD‐1 inhibitors.^12^ In addition, the higher tumor mutational burden (TMB) rates also predict the better efficacy of immune‐checkpoint inhibitors.^10^, ^13^, ^14^ Despite of these clinical successes, only a minority of CRC patients respond to PD‐1 blockade, of which the underlying mechanisms are not fully understood, highlighting the need for a better understanding of the tumor microenvironment of CRC.

In this study, we performed scRNA‐seq analysis in 12 treatment‐naïve CRC patients at cancer stages I–IV harboring distinct driver mutations and tumor mutational burden determined by whole exome sequencing (WES). We focused on immune populations and found tumor mutational burden states of human CRC contribute to distinct immune profile patterns. Further, we comprehensively profiled T‐, B‐, and myeloid‐cell subpopulations and their crosstalk with non‐immune cells. Akin to scRNA‐seq, single‐cell ATAC‐sequence (assay for transposase‐accessible chromatin using sequencing) enables high‐resolution profiling of cellular composition and heterogeneity and can generate additional information about chromatin accessibility landscape. We corroborated our findings by scATAC‐seq analysis in human CRC and inferred transcription factors that govern gene regulatory networks. Altogether, our datasets provide a resource for using defined clusters and states of immune cell subpopulations for both diagnostic and therapeutic research purpose for human colorectal cancer.

## 2 RESULTS

### 2.1 Profiling single‐cell transcriptomes and chromatin accessibility landscape of human CRC

**FIGURE 1:**
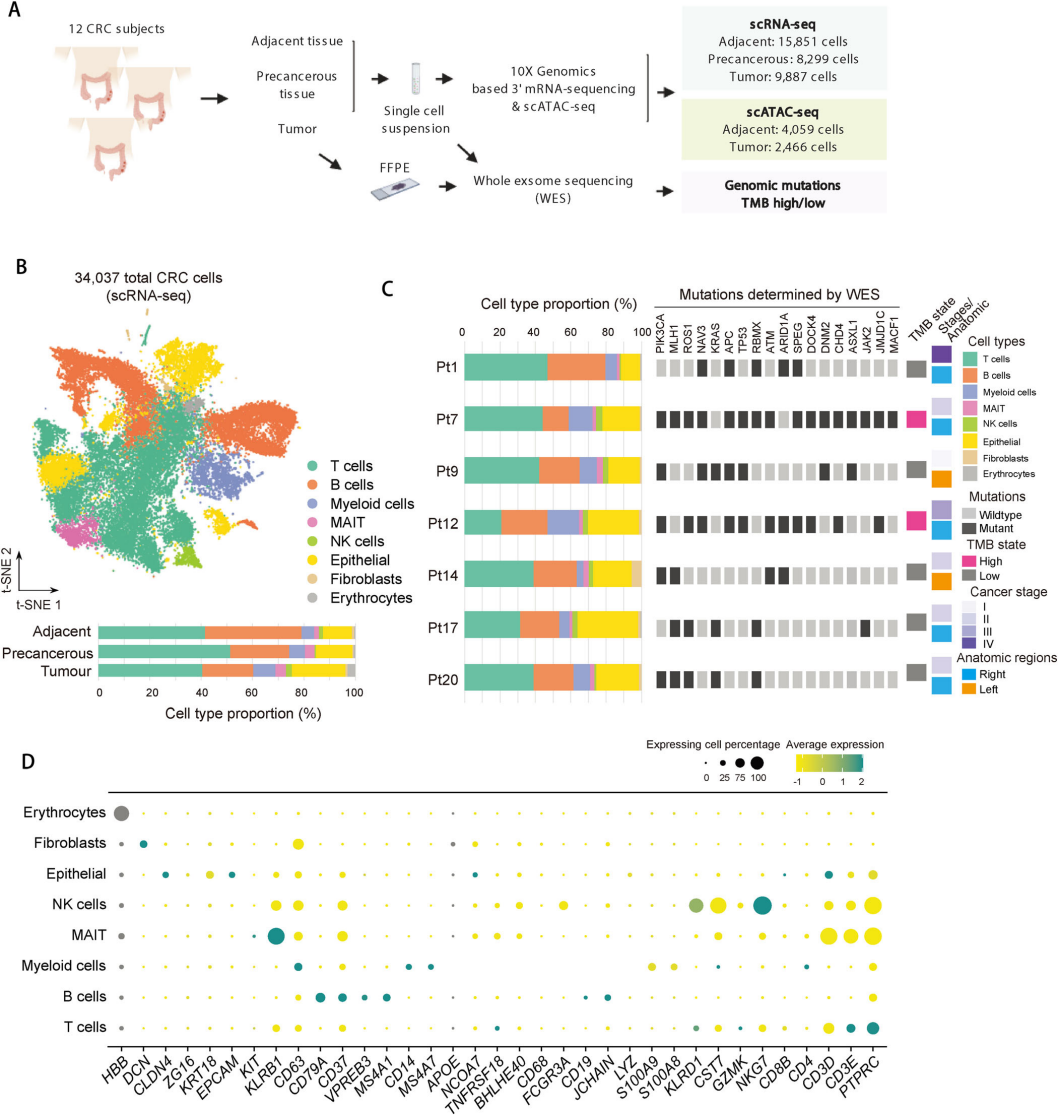
Single‐cell RNA‐seq profiles of the human CRC. (A) Schematic of the experimental design for scRNA‐seq of human CRC. FFPE, formalin‐fixed paraffin‐embedded tissue specimens. (B) *t*‐SNE plot of 34,037 cells from 12 CRC patients showing eight major cell types (top). Bar plot of cell proportions in adjacent, precancerous, and tumor tissues (bottom). MAIT, mucosal‐associated invariant T (MAIT) cells. (C) Proportions of the major cell types, the clinical information and mutations for individual samples are shown. Mutations were detected by whole exome sequencing (WES). Colors denote corresponding clusters. (D) Dot plot showing average expression of representative marker genes of major cell clusters of integrated human CRC data. Dot size represents proportion of cells

To elucidate the cellular landscape of microenvironment of human colorectal tumors, we analyzed a total of 34,037 CRC single cells generated by droplet‐based single‐cell RNA sequencing (Figure 1A,B). The cells were obtained from paired adjacent normal, precancerous and primary tumor tissues of 12 treatment‐naïve patients at cancer stages I–IV that were confirmed by pathological examination of paired biopsies (Table S1). In addition, we performed WES on tumor tissues of seven patients that were accessible, and identified the cohort harbored most of the canonical mutations detected in CRC, such as *PIK3CA, MLH1, ROS1, KRAS, APC, TP53, PIK3CG, JMJD1C, ASXL1, CHD4*, and *JAK2* mutations (Figure 1C). Of these seven subjects, patient numbers 7 and 12 (Pt7 and Pt12) harbored high mutational rates and were categorized as TBM high, while the others were classified as TMB low (Figure 1C).

Using graph‐based clustering to partition the cells and *t*‐distributed stochastic neighbor embedding (*t*‐SNE) for visualization,^15^ we integrated data from three tissue types (adjacent, precancerous, and tumor) and identified major cell populations including T cells, B cells, myeloid cells, mucosal associated invariant T (MAIT) cells, natural killer (NK) cells, epithelial cells, fibroblasts, and erythrocytes across all three tissue types (Figure 1B) by examination of canonical marker genes (Figure 1D). Erythrocyte contamination was removed for following analysis. Notably, all the major cell types were retrieved in distinct tissues or patients (Figure 1B–C). The samples showed a remarkable heterogeneity that is associated with different cancer stages (I–IV), tissues (adjacent, precancerous, and tumor), and tumor mutational burden (Figure S1).

**FIGURE 2:**
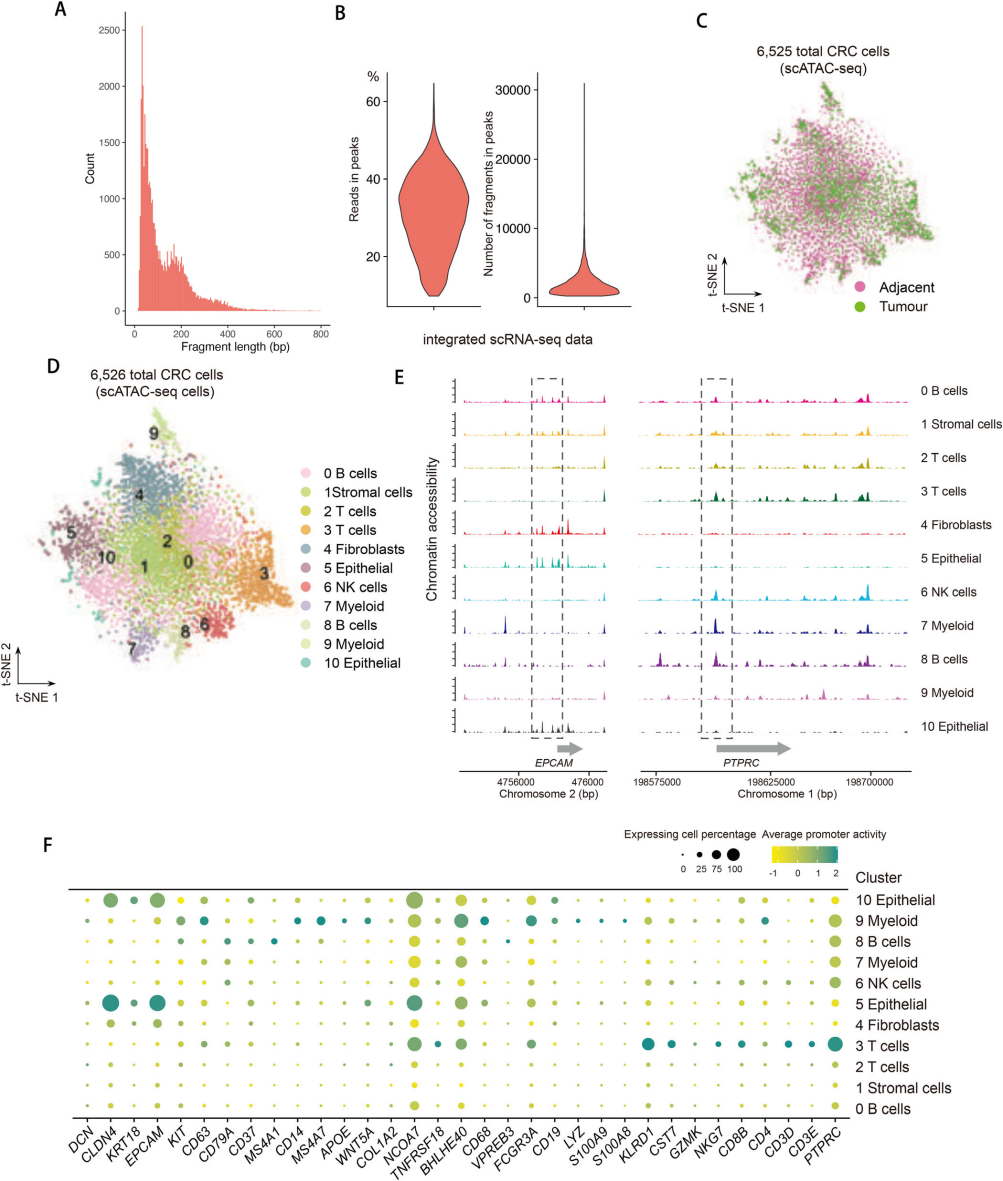
Single‐cell ATAC‐seq profiles of the human CRC. (A and B) Quality control of scATAC‐seq data. Plot showing the fragment length periodicity of signal from all single cells (A) and the proportion of all fragments that fall within ATAC‐seq peaks (B, left), total number of fragments in peaks (B, right). (C) *t*‐SNE plots showing scATAC‐seq data of human CRC cells color‐coded by adjacent and tumor tissues. (D) *t*‐SNE plots showing scATAC‐seq data of human CRC cells. (E) Aggregated ATAC‐seq tracks of individual clusters of genomic regions of indicated locus. (F) Dot plot showing average promoter activity of representative marker genes calculated based on scATAC‐seq signal. Dot size represents proportion of cells

To further explore the underlying molecular mechanisms that shape the tumor immune microenvironment, we performed single‐cell assay for transposase accessible chromatin using sequencing (scATAC‐seq) to profile the epigenetic landscape on 6526 CRC cells sampled from the same adjacent and tumor tissues for above scRNA‐seq from six patients (Pt7, Pt9, Pt12, Pt14, Pt17, and Pt20) (Figures 1A and 2). We employed term frequency‐inverse document frequency (TF‐IDF) normalization and singular value decomposition (SVD) for dimensional reduction to weight important peaks that enables detection of hidden subpopulations.^16^ This analysis enabled us to identify 11 clusters (Figure 2D). We noted that RNA‐based classification did not perfectly match the chromatin accessibility based dimensionality reduction, which is consistent with our previous findings of mismatch when pairing scATAC‐seq with scRNA‐seq clusters.^16^ To interpret the scATAC‐seq data, we performed cross‐modality integration and transferred cell type annotations from scRNA‐seq clusters to scATAC‐seq data by identifying cluster‐specific peaks and assigned genes that were proximal to such peaks (within 2 kb to TSSs). The aggregated reads from each cluster exhibited open chromatin loci of *PTPRC* (encoding *CD45*) and *EPCAM* in immune and non‐immune cells, respectively, which is consistent with their RNA expression in individual clusters (Figure 2E,F).

### 2.2 Tumor‐specific Tregs and highly proliferative exhausted T cells identified in human CRC

**FIGURE 3:**
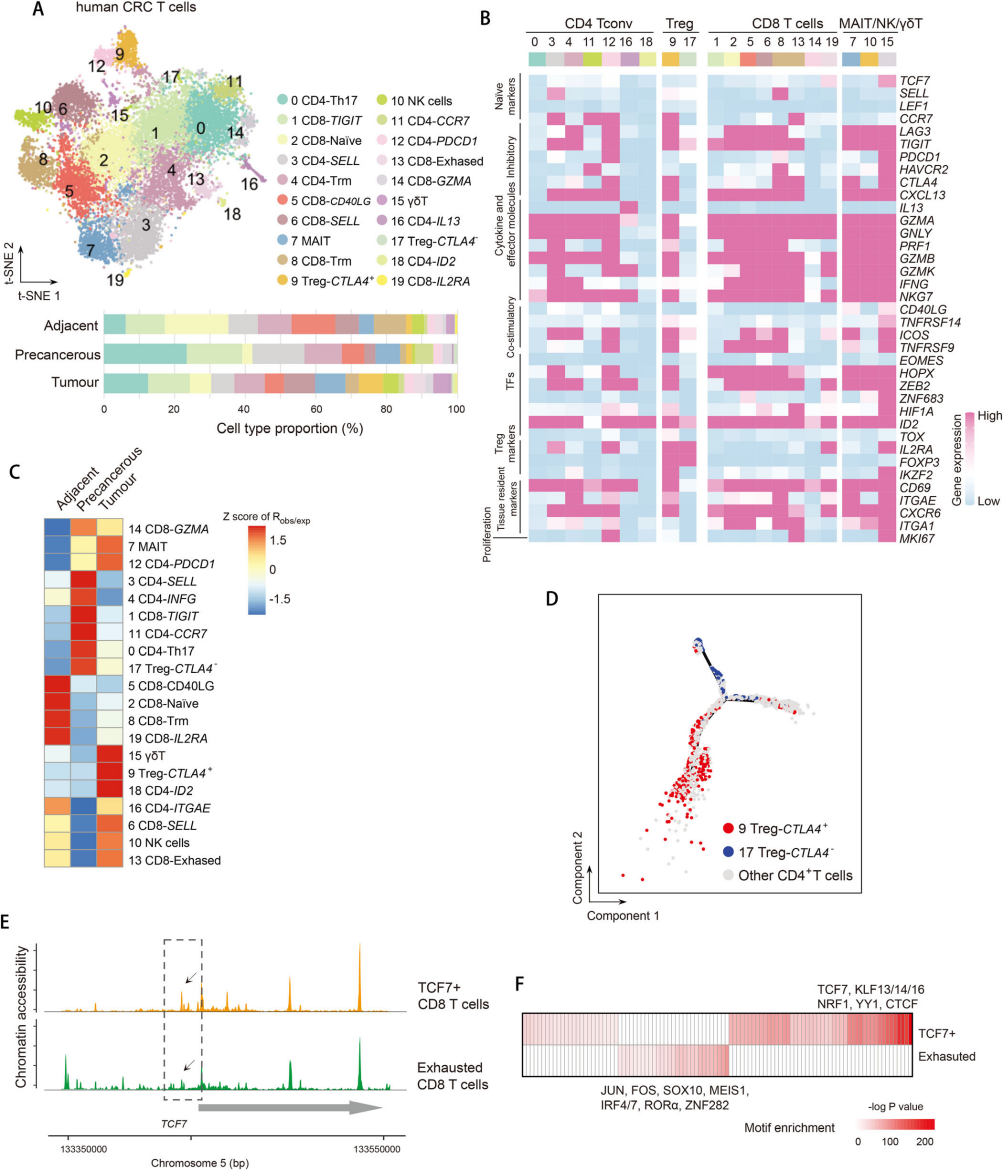
Presence of activated tumor Tregs, proliferative exhausted T cells in human CRC. (A) *t*‐SNE plot of T cells color‐coded by cell type and annotated by cluster numbers (top). Bar plot of cell proportions in adjacent, precancerous and tumor tissues (bottom). (B) Heatmap showing average expression of selected T‐cell function‐associated genes in each cell cluster. (C) Tissue preference of each cluster estimated by observed‐to‐expected ratio (*R*_obs/exp_). The *R*_obs/exp_ was *z*‐score transformed. (D) *t*‐SNE plots showing pseudo‐time paths of all CD4^+^ T cells. *CTLA4^+^* and *CTLA4^–^* Treg cells are denoted in red and blue, respectively, and other cells are in grey. (E) Genomic region of indicated locus showing ATAC‐seq tracks of aggregated single cells of *TCF7* ^+^ and exhausted T cells. (F) Heatmap showing the enrichment of transcription factor motifs in *TCF7* ^+^ and exhausted T cells. *p*‐Values were calculated from the hypergeometric distribution

To unravel the intrinsic subpopulations and potential functions, we subset and performed unsupervised re‐clustering of each major cell types. T lymphocytes (including T and MAIT cells) and NK cells comprised the largest immune cell cluster across adjacent normal, precancerous, and tumor tissues in our datasets. We re‐clustered 16,397 single T and NK cells and identified 20 subpopulations, including a total of eight *CD8^+^* and nine *CD4^+^* T‐cell clusters, one NK cell cluster, one MAIT cluster, and one γδT cell cluster (Figure 3A,B). We identified clusters according to previously defined immune markers in colon tissues^7^, ^8^, ^17^ as well as DE (differentially expressed) genes in each cluster (Figure S2A and Table S4). To statistically quantify tissue enrichment of each subpopulation, we performed χ^2^‐test and measured the ratio of observed to expected cell numbers (*R*_obs/exp_) (see Methods). By comparing tissue distribution preference, we observed both NK cells and MAIT cells were predominantly enriched in CRC tumor tissues (Figure 3C). We found the tumor infiltrating MAIT cells expressed genes related with activation and exhaustion, such as pro‐inflammatory cytokine TNFα, *CTLA‐4, HAVCR2*, Granzyme A, and Granzyme B, suggesting their polyfunctionality in response to bacteria and/or metabolites present in the tumor microenvironment in CRC (Figure 3B).

For *CD4^+^* T cells, we identified two clusters of Th1 cells (expressing *IFN‐γ*) (clusters 3 and 4), one cluster of Th2 cells expressing IL13 (cluster 16), two clusters of *FOXP3^+^* Treg cells (clusters 9 and 17), and exhausted *CD4^+^* T cells (cluster 12). For *CD8^+^* T cells, we designated clusters 2, 5, and 6 as cytotoxic T cells (highly expressing *GZMK PRF1, GZMA*, and *GZMB*, and showing low expression of exhaustion markers such as *PDCD1, CTLA4*, and *HAVCR2*), and identified naïve *CD8^+^* T cells (clusters 8 and 19; expressing *TCF7* and *SELL*) and exhausted *CD8^+^* T cells (cluster 6; *CD8^+^* T_EX_) (Figure 3B). Further, clusters 4 and 8 highly expressed *CD69, ITGAE*, and *CXCR6*, and were therefore designated as tissue‐resident memory T cells (T_RM_). Two Treg clusters can be distinguished by the expression of *CTLA4* (Figure 3B). The *CTLA4^+^* Treg cluster (cluster 9) was present mostly in tumors, whereas the *CTLA^–^* Treg cluster was enriched in adjacent tissues (Figure 3C). Differentially expressed gene analysis revealed two distinct transcription programs by showing *CTLA4^+^* Treg highly expressed *IL32, KLF6, HLA‐DRB1, ETS1, IL7R, KMT2E, ISG20, TCF25, TIGIT* and *CD69*, whereas *TOX2* and *ANKRD17* were enriched in *CTLA4^–^* Tregs (Figure S2B). Alignment of all T cells along the potential developmental trajectories also corroborated distinct transcriptome programs of *CTLA4^+^* and *CTLA^–^* Treg cells, indicating their different origination and cell function (Figure 3D and Figure S2C). We found both *CD4^+^* and *CD8^+^* T_EX_ cells in CRC highly expressed *MKI67* (Figure 3B). The proliferative status was positively correlated with differentiated exhausted states of T_EX_ during chronic infections.^18^ Intriguingly, although *CD8^+^* T_EX_ cells were in exhaustion state, they still expressed comparable levels of effector molecules such as *IFNG, GZMB, GZMH*, and *PRF1* as other *CD8^+^* subsets (Figure 3B).

We then analyzed scATAC‐seq data in T cells and identified nine subpopulations based on promoter accessibility of marker genes (Figure S2D,E). The T‐cell subpopulations of scATAC‐seq also showed unequal preference in adjacent and tumor tissues; for example naïve T cells (CD8‐*TCF7* and CD4‐*CCR7*) were found predominantly in adjacent tissues, whereas exhausted T cells (CD8‐*HAVCR2*) were enriched in the normal tumors (Figure S2F). Consistently, the accumulated cluster‐specific chromatin accessible peaks of *TCF7* locus were decreased in exhausted T cells comparing with that in *TCF7^+^* cells (Figure 3E), which is reminiscent of previous study of TCF1 (encoded by *TCF7*) patterns revealed by bulk ATAC‐seq.^19^ We then inferred enriched TF binding sites within accessible chromatin regions in *TCF7* ^+^ and exhausted T cells. We found a group of KLF family genes (KLF13/14/16) as well as TCF7 enriched in *TCF7* ^+^ cells, which suggested such genes may play a role in maintaining T‐cell stemness (Figure 3F). SOX10, MEIS1, RORα, and ZNF282 were enriched in exhausted cells, which requires further study.

### 2.3 Tumor mutational burden associated T‐cell subpopulations in human CRC

**FIGURE 4:**
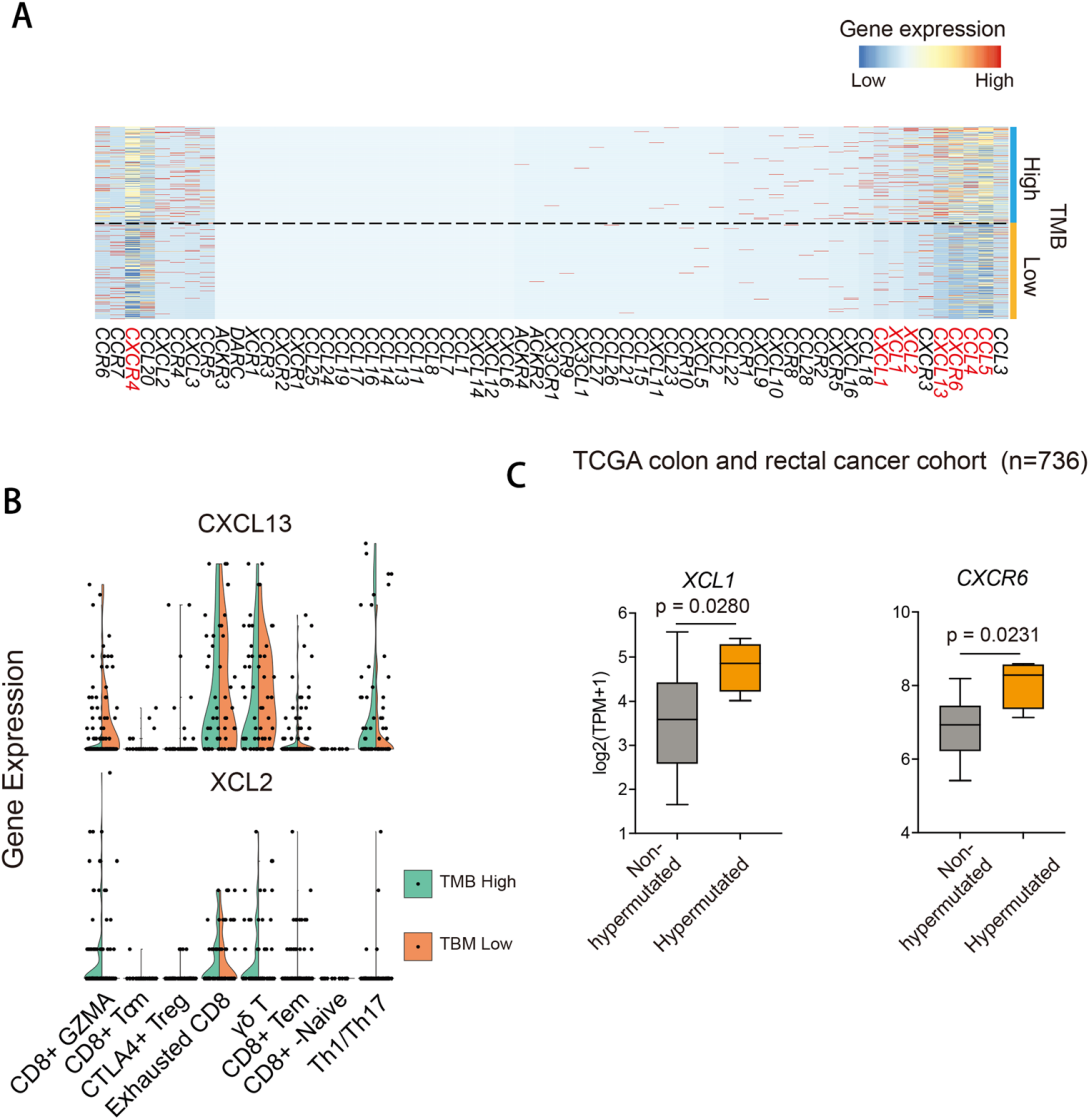
Tumor mutational burden‐related T‐cell heterogeneity. (A) Heatmap showing gene expression of chemokine genes in T cells of TMB high and low samples. (B) Violin plot showing gene expression of indicated genes in T cells of TMB high and low samples. The dots represent outliers. (C) Normalized expression of indicated genes in TCGA COADREAD data. Hypermutated and nonhypermutated samples are separated based on genome‐wide gene mutation rates. Boxplot showing the first quartile, median, and the third quartile. Whiskers extend 1.5 times of the interquartile range. *p*‐Value was calculated using Student's *t*‐test

TMB has been reported to predict response to immune checkpoint inhibitors in colorectal cancer as a biomarker independent of microsatellite instability status.^13^, ^14^ We identified differentially expressed genes associated with TMB classification and focused on chemokines (Figure 4A). We found the abundance of Th1/Th17 cells expressing CXCL13 was increased in TMB high in comparison with TMB low subjects (Figure 4A,B). This finding is concordant with previous study that a cluster of *CXCL13* ^+^ Th1‐like cells were preferentially enriched in microsatellite‐instable tumor that usually corresponds to TMB high and shows favorable responses to immunotherapy.^8^, ^10^ In addition, we identified that lymphotactin 1 and 2 (XCL1 and XCL2), the ligand of chemokine XC receptor 1 and 2 (*XCR1* and *XCR2*), were highly expressed in *CD8^+^* CTL and T_EX_ cells in TMB high than in TMB low tumors (Figure 4A,B). We subsequently examined the association of gene signatures of TMB states with DNA mutation rates in colorectal cancer from the Cancer Genome Atlas (TCGA COADREAD cohort). We found that remarkably higher expression of *CXCR6* was associated with hypermutated subjects comparing with nonhypermutated ones (Figure 4C). In addition, analysis of *XCL1* indicated increased expression in hypermutated subjects compared to nonhypermutated ones (Figure 4C). *XCL1* is expressed in activated T cells and involved in antigen presentation of dendritic cells (DCs),^20^ and thus the higher expression of *XCL1* likely contributes to activating antitumor T‐cell responses by recruiting DCs.

### 2.4 Clustering myeloid cells reveals dichotomous function of tumor‐associated macrophages

**FIGURE 5:**
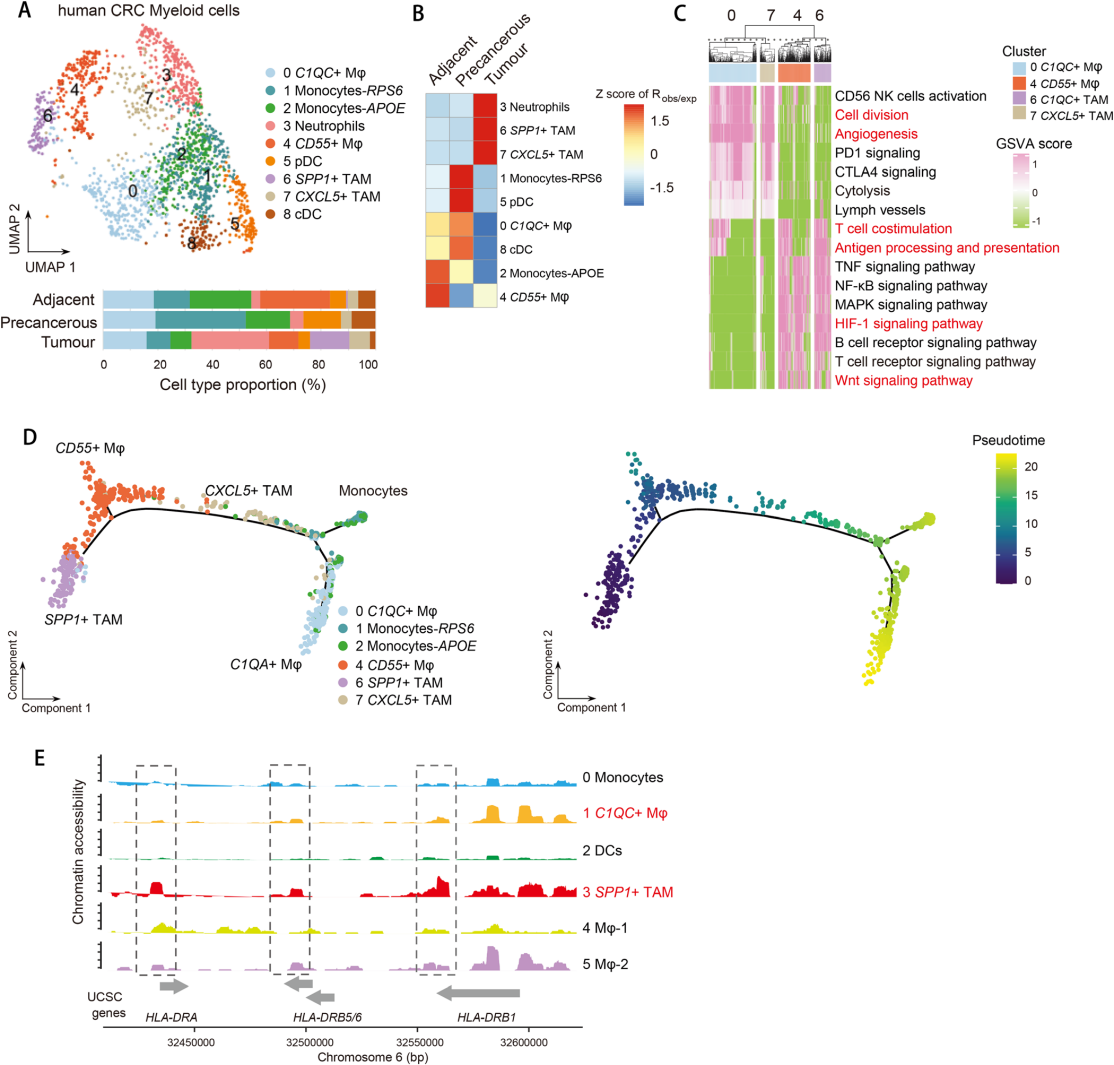
Tumor‐associated macrophage subpopulations in CRC. (A) UMAP plot of myeloid cells (top). Bar plot of cell proportions in adjacent, precancerous, and tumor tissues (bottom). (B) Tissue preference of each cluster estimated by observed‐to‐expected ratio (*R*_obs/exp_). The *R*_obs/exp_ was *z*‐score transformed. (C) Pathway enriched in *C1QC* ^+^ and *SPP1* ^+^ TAMs, and *CXCL5* ^+^ and CD55^+^ macrophages. Prominent pathway terms are highlighted in red. Mean score of GSVA was *z*‐score transformed. (D) *t*‐SNE plots showing pseudo‐time paths of monocytes, *C1QC* ^+^ and *SPP1* ^+^ TAMs, and *CXCL5* ^+^ and CD55^+^ macrophages. (E) Genomic region of *HLA‐DRB* locus showing ATAC‐seq tracks of aggregated single cells of myeloid cells

Myeloid cells partitioned into nine subpopulations (Figure 5A, Figure S3A, and Table S2). Using bioinformatical criteria to include cells with low transcript counts that could be inadvertently excluded when using the common data filtering standard,^21^ we identified hyperinflammatory neutrophils enriched in tumors comparing with adjacent and precancerous tissues (Figure 5B). DCs, including plasmacytoid DCs (pDCs) and conventional DCs (cDCs), play a central role in cancer immunity by processing tumor antigen and activating T cells.^22^, ^23^, ^24^ In our dataset, pDCs and cDCs were less enriched in tumors comparing with other tissues (Figure 5B), which suggests antigen presentation ability and T‐cell activation is compromised due to attenuation of DC enrichment and may partially explain the dysfunctional T‐cell immunity of tumors.

We identified four macrophage (Mφ) subpopulations, of which *C1QC^+^* Mφ and *CD55^+^* Mφ (clusters 0 and 4) showed preferential enrichment in adjacent rather than in tumor tissues. *SPP1^+^* and *CXCL5^+^* Mφ (clusters 6 and 7) clusters were enriched in tumor tissues and therefore were designated as tumor‐associated macrophages (TAMs). Surprisingly, we found *SPP1^+^* TAMs (cluster 6) had mixed phenotypes of proinflammatory and anti‐inflammatory functions, whereas *CXCL5^+^* TAMs (cluster 7) showed proinflammatory phenotypes (Figure S3B). We performed gene set variation analysis (GSVA) and found *SPP1^+^* TAMs and *CD55^+^* Mφ significantly enriched “antigen processing and presentation” and “T‐cell co‐stimulation” pathways, while *CXCL5^+^* TAMs and *C1QC^+^* Mφ enriched “angiogenesis” pathways (Figure 5C). This suggests that such diverse macrophage subsets not only orchestrate immune responses but also control tumorigenesis via tumor angiogenesis. In addition, we found *SPP1^+^* TAM exhibited enrichment of Wnt signaling pathway, which may support tumor growth^25^ (Figure 5C). By ordering monocytes and macrophages to reconstruct pseudo‐time trajectories using Monocle 2 algorithm,^26^ we observed a clear directional flow that monocytes bifurcated to two branches of *SPP1^+^* TAMs and *C1QC ^+^* Mφ (Figure 5D), suggesting distinct cellular differentiation paths of these two subpopulations. In concordance with the enriched antigen processing and presentation pathways in *SPP1^+^* TAMs, we found elevated expression of MHC class II genes such as *HLA‐DRB5*, *HLA‐DQA1*, and *HLA‐DQB1* comparing in *SPP1^+^* TAMs with *CXCL5^+^* TAMs (Figure S3C). Our scATAC‐seq analysis of myeloid cells in adjacent and tumor tissues was also able to recover the *SPP1^+^* TAMs (cluster 3) (Figure S3D–F). In line with the dichotomous function revealed by scRNA‐seq, both proinflammatory and anti‐inflammatory genes exhibited opening promoter activities in *SPP1^+^* TAMs (Figure S3G). Consistent with the antigen processing and presentation ability, the *HLA‐DRA* and *HLA‐DRB* loci were more open compared with other clusters (Figure 5E). To further explore the molecular mechanism that regulates *SPP1^+^* TAMs, we used scATAC‐seq data to identify transcription factor motifs that become accessible due to nucleosome eviction and/or chromatin remodeling using R package chromVar.^27^ We found a strong enrichment of Krüppel‐like factor family (KLF) KLF14 and KLF16 in *SPP1^+^* TAMs, while the ETV family, ELK4, ZBTB7A, YY1, and RUNX3 were significantly enriched in *C1QC^+^* Mφ (Figure S3H). Taken together, our analysis delineates the TAM subpopulation with mixed proinflammatory and anti‐inflammatory function, further revealing that TAMs are more phenotypical and functionally diverse than conventional M1 and M2 classification, which is reminiscent of recent studies.^5^, ^9^

### 2.5 Attenuated B‐cell antigen presentation in tertiary lymphoid structures in human CRC

**FIGURE 6:**
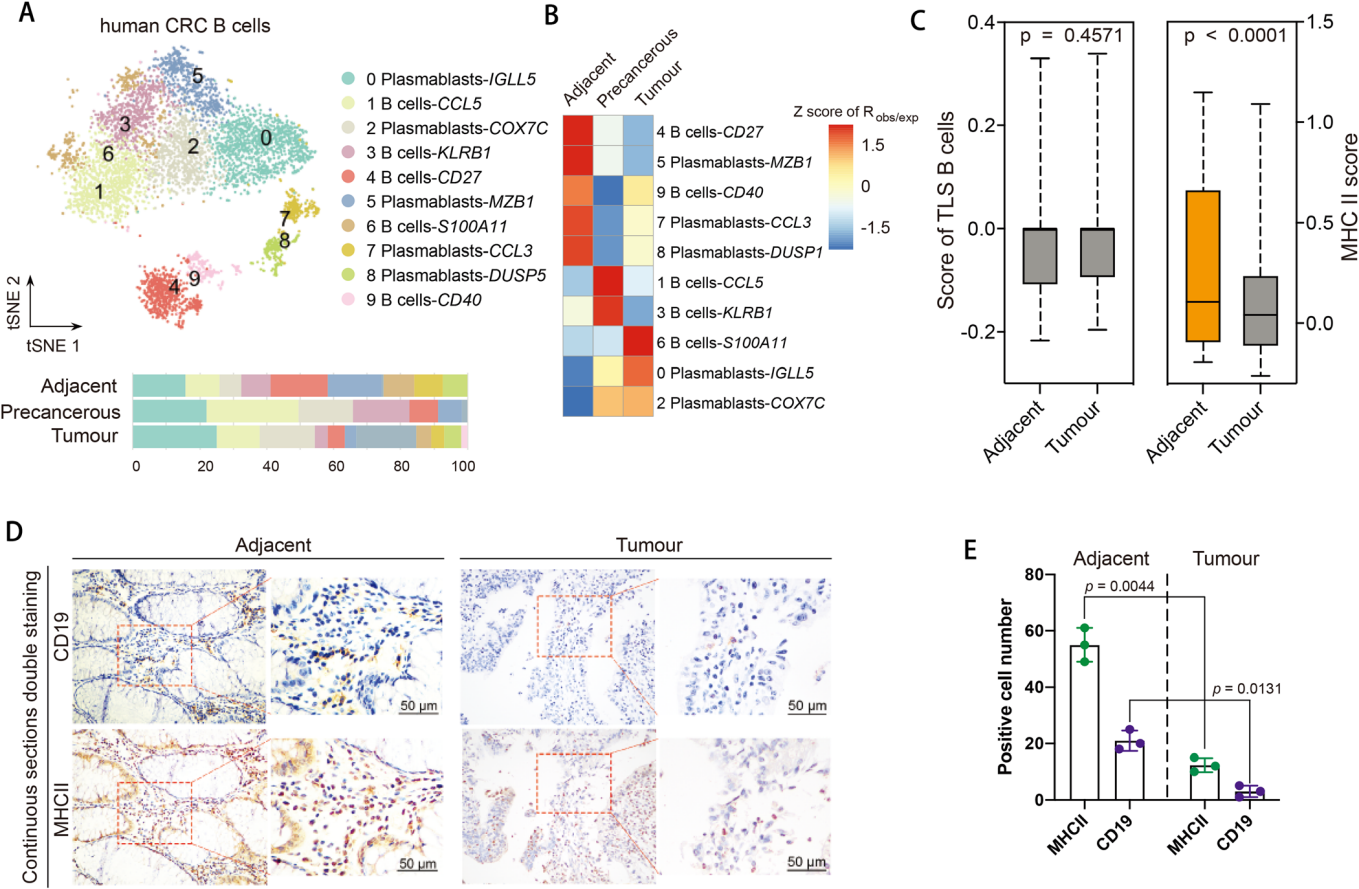
Tumor‐associated B‐cell subpopulations in CRC. (A) *t*‐SNE plot of B cells (top). Bar plot of cell proportions in adjacent, precancerous, and tumor tissues (bottom). (B) Tissue preference of each cluster estimated by observed‐to‐expected ratio (*R*_obs/exp_). The *R*_obs/exp_ was *z*‐score transformed. (C) Tertiary lymphoid structure score (left) and MHC class II gene score (right) in adjacent and tumor tissues. Boxplot showing the first quartile, median, and the third quartile. Whiskers extend 1.5 times the interquartile range. *p*‐Value was calculated using Student's *t*‐test. (D and E) IHC staining (D) and statistical bar plot (E) of CD19 and MHCII in adjacent and tumor tissues. The serial sections of the same specimen are used to co‐localize CD19 with MHCII in the same region. IHC staining pictures are exemplified by patient Pt12. Bar plots represent results of Pt9, Pt12, and Pt17. The scale bar represents 100 μm (left panels) and 50 μm (right panels). *p*‐Value was calculated using Student's *t*‐test

**FIGURE 7:**
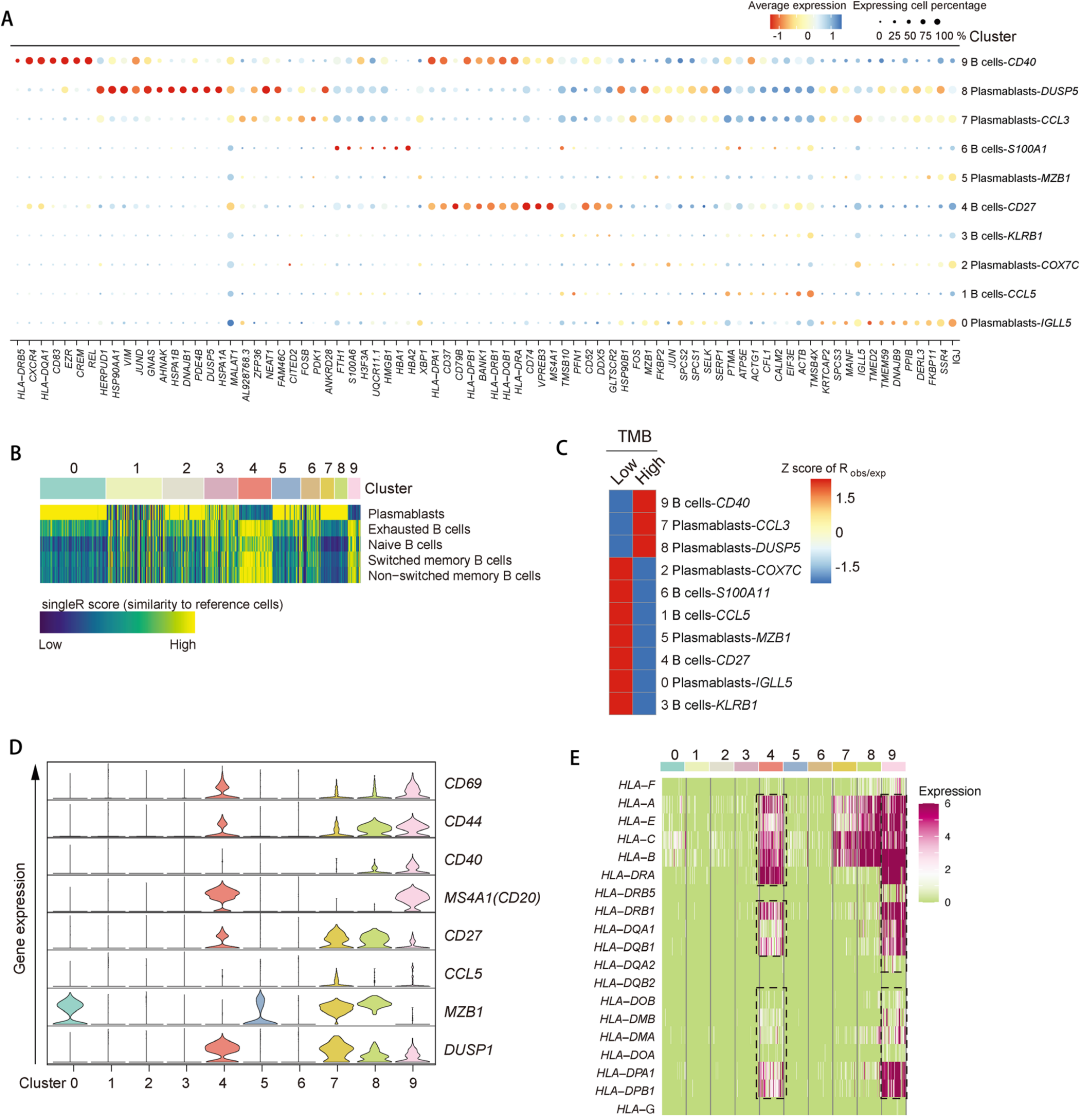
Single‐cell RNA‐seq of B cells of the human CRC. (A) Dot plot showing differentially expressed genes in each cluster. Dot size represents proportion of cells. (B) Heatmap showing similarity score of B‐cell clusters comparing with reference cells. The numbers indicate the same clusters as clusters in (A). (C) Tissue preference of each B‐cell cluster estimated by observed‐to‐expected ratio (*R*_obs/exp_). The *R*_obs/exp_ was *z*‐score transformed. (D) Violin plots showing gene expression of marker genes across B‐cell subpopulations. The *y*‐axis was log‐scaled. Numbers indicate the same clusters as clusters in (A). (E) Heatmap showing gene expression of MHC genes in B cells. Numbers indicate the same clusters as clusters in (A)

The overall shrinkage proportion of B and plasma cells in tumor tissues suggests their potential roles in shaping the tumor microenvironment (Figure 1A). We identified five subpopulations of B cells and five plasma cell subpopulations with marker‐based annotations, identification of cluster‐specific differentially expressed (DE) genes and by comparing with reference cells via SingleR^2^ (Figures 6A and 7A,B and Table S3). In comparison with adjacent and precancerous tissues, we found a significant reduction of *CD40^+^, CD27^+^, KLRB1^+^*, and *CCL5^+^* B cells (clusters 4, 9, 1, and 3, respectively) and *MZB1^+^, DUSP1^+^*, and *CCL3^+^* plasma cells (clusters 5, 7, and 8) in tumor tissues (Figure 6B). Clusters 4, 7, 8, and 9 coexpressed *CD44* and *CD69*, which are the hallmark genes of tissue residency of B cells in lung,^28^ and we therefore designated these subpopulations as tissue‐resident memory B cells in colon mucosal (Figure 7C). We observed underrepresented proportion of tissue‐resident memory B cells in tumor tissues, suggesting a systemic change in the B‐cell immune microenvironment (Figure 6B). By identifying DE genes in each cluster (Table S3), we observed increased expression of MHC class II genes in *CD40^+^* and *CD27^+^* B cells (Figure 7D). Recently, it was reported that proliferative B‐cell signatures were enriched in human tumors that respond to immunotherapy but not in the nonresponding ones, and such B cells localize within tertiary lymphoid structure (TLS) in several types of cancers including melanoma and soft tissue tumor.^29^, ^30^, ^31^ Hence, we were promoted to evaluate the TLS score by the examination of marker gene expression including *CCL19, CCL21, CXCL13, CCR7, SELL, LAMP3*, and *CXCR4* and observed similar TLS genes score in tumors comparing with adjacent tissues, which implied the tertiary lymphoid structures in CRC tumor was intact (Figure 6C). We then assessed the proliferation and antigen presentation ability of *CD19^+^* and *CD20^+^* B cells. We found their proliferation states were higher and more disperse in tumor tissues comparing with adjacent and precancerous tissues, but the MHC class II genes score that may indicate antigen presentation ability was lower (*p* < .0001, Student's *t*‐test) (Figures 6C and 7E). Consistently, the IHC staining of CD19 and MHC class II proteins showed that the number of HLA‐DPB1^+^ and CD19^+^ cells were significantly decreased in tumor comparing with adjacent regions (*p* = .0004, paired Student's *t*‐test) (Figure 6D,E). Overall, our analysis evaluated B‐cell immunity in human CRC and revealed the attenuated antigen presentation and diminished antitumor immunity capacity of *CD40^+^* and *CD27^+^* B cells in tumor.

### 2.6 Tumor immune microenvironment of CRC is affected by non‐immune cells

**FIGURE 8:**
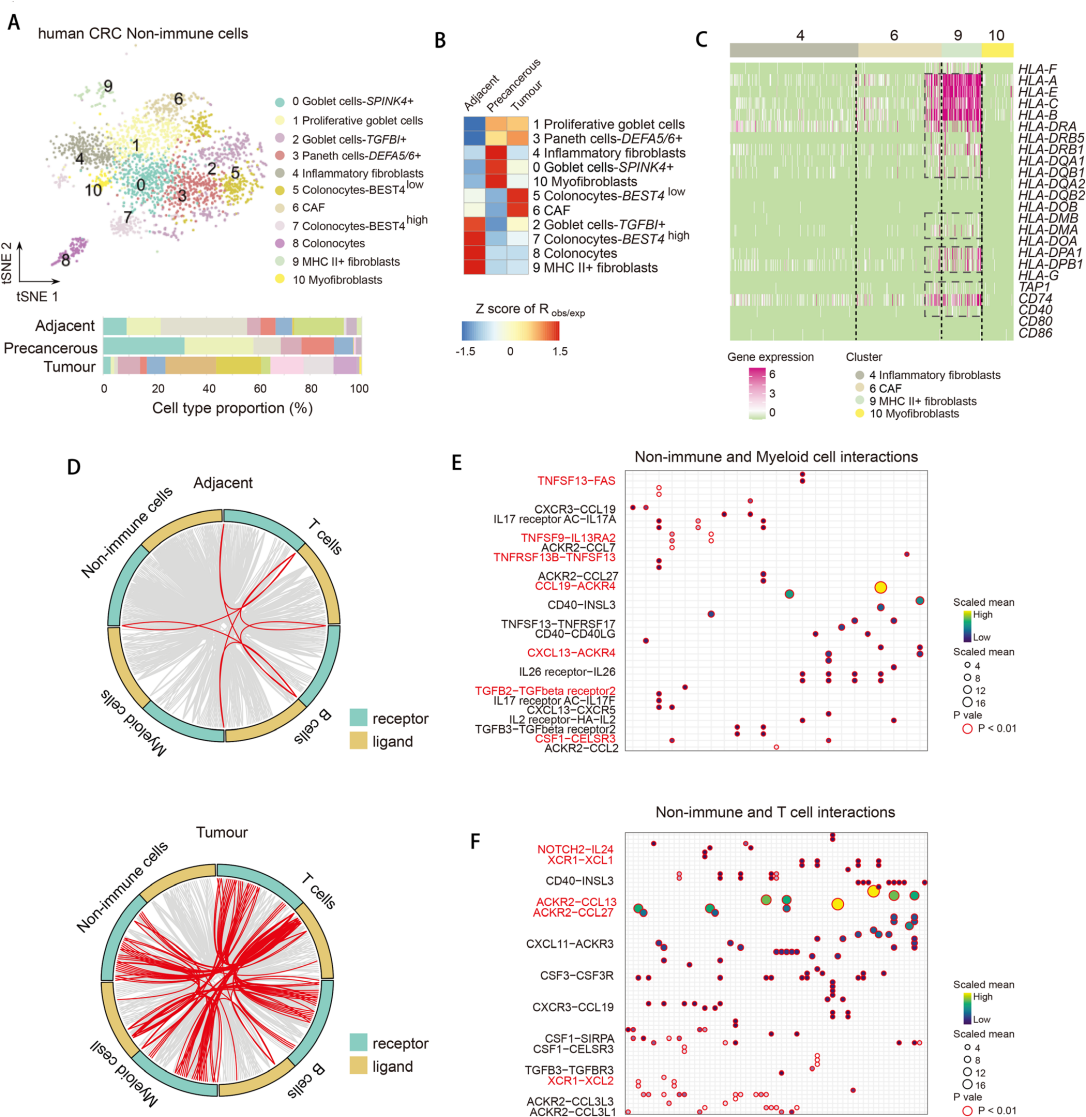
MHCII expression of cancer‐associated fibroblast subpopulations and putative cell–cell communications between non‐immune and immune cells. (A) *t*‐SNE plot of non‐immune cells (top). Bar plot of cell proportions in adjacent, precancerous, and tumor tissues (bottom). (B) Tissue preference of each cluster estimated by observed‐to‐expected ratio (*R*_obs/exp_). The *R*_obs/exp_ was *z*‐score transformed. (C) Heatmap showing expression of selected MHC and antigen presentation related genes in each cell cluster. (D) Circos plot showing the interactions between ligands and receptors across cell types in adjacent (top) and tumor tissues (bottom). The interactions between non‐immune and immune cells are highlighted in red, other interactions are in grey. (E and F) Heatmap showing ligands and receptor interaction pairs in tumor tissues between non‐immune and myeloid (E) or T cells (F)

We clustered non‐immune cells into 11 subpopulations according to cluster‐specific DE genes (Figure 8A, Figure S4A, and Table S5). High proliferative goblet cells expressing *MKI67* and metaplastic paneth cells expressing antimicrobial α‐defensins (*DEFA5* and *DEFA6*) were enriched in precancerous and tumor tissues comparing with adjacent tissues (Figure 8A), suggesting they might play a role in tumorigenesis. Colonocytes highly expressing *BEST4* were enriched in adjacent tissues, whereas *BEST4* ^low^ colonocytes exhibited tumor preference (Figure 8B). Four fibroblast subpopulations were identified (clusters 4, 6, 9, and 10), and cluster 6 was designated as cancer‐associated fibroblasts (CAFs) as it was enriched in tumors (Figure 8B). The CAFs were classified into myofibroblasts and inflammatory fibroblasts subtypes based on high expression of either α‐SMA or cytokines and chemokines.^32^ We found a subpopulation of fibroblasts highly expressed MHC II class genes and antigen presentation machinery such as *CD74* and *TAP1* (Figure 8C and Figure S4B). We therefore hypothesized such novel fibroblast subpopulation may have the capacity to present antigen to T cells. However, MHC II^+^ fibroblasts did not express costimulatory genes such as *CD40, CD80*, and *CD86* for T‐cell activation (Figure 8C), suggesting their incapability to support full T‐cell activation and thereby leads to T‐cell tolerance or anergy. Reconstruction of cell differentiation pseudo‐time revealed MHC II^+^ fibroblasts, inflammatory fibroblasts, and CAFs have different origination and cellular functions (Figure S4C), suggesting the loss of costimulatory molecules of fibroblasts in tumors may contribute to the maintenance of T‐cell exhaustion.^33^

Cell‐to‐cell communication, which is partially mediated by ligand (surface or secreted) and receptor interactions, is important for immune cells to function in both normal and tumor tissues. We focused on the crosstalk between non‐immune and immune cells and interrogated ligand and receptor expression of single cells to predict cell‐to‐cell communication in adjacent and tumor tissues using R package CellTalker.^34^ We found strikingly massive expansion of non‐immune and immune cells communications in tumor comparing with adjacent tissues (Figure 8D), suggesting the active roles of non‐immune cells in shaping tumor immune microenvironment. We further inferred receptor and ligand interactions in adjacent and tumor tissues using CellPhoneDB packages^35^ (Figure S5A,B). Closer examination of non‐immune and myeloid cell interactions in tumor revealed TNF superfamily and receptors such as *TNFSF13−FAS* (Figure 8E), suggesting signaling via TNF superfamily play an important role in regulating myeloid cells in TME. TNF and Fas interactions have been reported to regulate macrophage polarization,^36^ however their functions in tumor remain unknown. Other interaction pairs involved *CSF1−CELSR3* and *CXCL13−ACKR4*. CSF1R signaling has been reported to control macrophage homeostasis.^37^ The interactions of non‐immune and T cells included *NOTCH2−IL24, XCR1−XCL1/2*, and *ACKR2‐CCL13/27* (Figure 8F). Of note, NOTCH signaling is required for T‐cell activation and effector function.^38^ The inference of cell–cell communications also allowed us to compare receptor and ligand interactions between immune lineages from a global perspective. For example, we found different chemokine and receptor interactions between B and T cells in adjacent and tumor tissues (Figure S5C,D). Our analysis provided a framework for assessing putative cell‐to‐cell communication to understand the function of non‐immune cells in shaping immune microenvironment in colorectal cancer.

## 3 DISCUSSION

In this study, we generated 34,037 single‐cell transcriptomes and chromatin accessibility landscape of 6525 single cells sampled from 12 CRC patients. Our large dataset allowed us to elucidate how heterologous cellular composition determines CRC microenvironment in individual patients. It is reported that CRC patients with high TMB respond better to the immune‐checkpoint blockade of PD‐1 but the ones with low TMB phenotypes do not. Single‐cell analysis of a cohort of CRC patients with MSI or MSS (microsatellite stability) identified that *CXCL13^+^BHLHE40^+^* Th1‐like cells were preferentially enriched in MSI.^8^ We performed single‐cell RNA‐seq on a cohort of CRC patients with high and low TMB to unravel their distinct immune cell composition. It is worth noting that WES analysis confirmed that all these patients in our cohort were MSS. Interestingly, we also identified *CXCL13* ^+^ T cells in high‐TMB tumors, which may play the similar function as in MSI tumors and be responsible for the high response rate to checkpoint blockade. In addition, we identified several TMB state‐related genes including *XCL1* and *2* family genes that were further confirmed through IHC staining, and significantly more samples retrieved from TCGA cohort. High TMB reflects high neoantigen loads at certain degree, and thereby tumor with higher TMB can induce stronger immune responses.^39^ *CXCL13* ^+^ and *XCL1/2^+^* T cells thus likely represent neoantigen‐reactive T cells. XCL ligand and receptor family genes are predominantly expressed in activated T cells and dendritic cells, mediate GPCR signaling, and induce efficient cytotoxic immunity.^40^, ^41^ It is plausible that XCL‐XCR signal serves as a niche cue to mediate antitumor immune response in high‐TMB tumors, which requires further study.

Currently, the immunotherapy for human CRC such as PD‐1 or CTLA‐4 blockade mainly leverages T‐cell function. Our detailed classification of tumor‐infiltrating T cells can provide molecular mechanism on how checkpoint blockade drugs affect T‐cell subpopulation. We identified two subpopulations of Tregs, that is, *CTLA4^+^* and *CTLA4^–^* Tregs in CRC, which are also identified in colon and non‐small‐cell lung cancer.^42^, ^43^ The presence of *CTLA4^+^* Tregs in tumors and *CTLA4^–^* Tregs in adjacent tissues is in line with observations that anti‐CTLA4 therapies may target *FOXP3^+^* Tregs to induce tumor rejection in melanoma.^44^ However, a recent contradictory result showed that *FOXP3^+^* Tregs were not affected in cancer patients treated with anti‐CTLA4 therapies.^45^ Hence, the clinical significance of such heterogeneity of Treg populations needs further characterization.

In this study, we identified nine distinct myeloid cell clusters in human CRC, suggesting that myeloid cells in tumor are more heterologous than previously appreciated.^21^ Importantly, we identified the polyfunctional tumor‐associated myeloid cells, namely *SPP1^+^* TAM subpopulations in CRC, whose functions do not fit the M1 and M2 polarization paradigm. In line with this, expanded *SPP1^+^* Mφ cluster was recovered and proposed to have both pro‐ and anti‐inflammatory features through scRNA‐seq analysis in a recent study.^5^ Moreover, another scRNA‐seq analysis in CRC also identified such TAMs and showed that *C1QC^+^* and *SPP1^+^* TAMs have inflammatory and angiogenic capacity, respectively.^9^ Targeting TAM subsets with CSF1R blockade may provide novel immunotherapy methods.^9^ However, as targeting CSF1R affects all macrophages, other strategies are therefore needed to target specific subsets.

So far, the chromatin accessibility landscapes of human CRC have not been investigated. We performed scATAC‐seq on human CRC samples and focused on the immune cells by inferring TFs that may play role in individual subpopulations by binding cognate motifs. Furthermore, we analyzed chromatin accessibility dynamics during T‐cell exhaustion, and observed the epigenetic changes of the *TCF7^+^* stem‐like to exhausted cells. Interestingly, when coupled promoter opening activity of scATAC‐seq data with gene expression of scRNA‐seq data, we observed unmatched ATAC:RNA pairs, which was observed in our previous studies and may suggest that the chromatin accessibility regulate targeting genes through long‐distance interactions such as enhancer–promoter interaction.^16^

In summary, our comprehensive characterization of immune cells from different cancer stages of colorectal cancer reveals the dynamic nature of immune and non‐immune cells. With the accumulation of more and more single‐cell transcriptional profiling of tumors from different studies, integration and comprehensive characterization of these data are necessary to gain the deeper insight to tumor microenvironment.^46^ Our data can be a valuable resource for further investigation to gain deeper biological insights that will lead to discovery of novel biomarkers and therapeutic targets for current immunotherapies for human colorectal cancer.

## 4 METHODS

### 4.1 Human sample acquisition

In this study, we analyzed 12 human primary CRC samples (Table S1). For Pt1, the fresh biopsies were obtained by colonoscopy, and single‐cell suspension was immediately prepared and subjected to scRNA‐seq analysis. For other samples, the tissues were obtained from patients undergoing surgical resection, and single‐cell suspensions were prepared and cryo‐preserved in 90% FBS/10% DMSO until further usage. The counterpart sections of all tumor tissues were reviewed by pathologists to confirm the diagnosis. This study was conducted with approval of the Ethics Committee of Sun Yat‐sen University Cancer Center and Zhongshan Hospital, Fudan University.

### 4.2 Sample dissociation

Human tumor or adjacent tissues were minced into small pieces of 1–2 mm in a petri dish on ice in the RPMI‐1640 medium (Invitrogen), and enzymatically digested in digestion buffer (25 mM HEPES [pH7.4], 0.15% Collagenase IV [m/v], 0.15% Collagenase II [m/v], 0.1% DNase [m/v], 5% FBS, 2 mM L‐glutamine, 5 mM CaCl2 in HBSS buffer with Ca^2+^Mg^2+^) for 45 min on a rotor at 37°C. The dissociated cells were passed through 70 μm and 40 μm cell‐strainer (BD) sequentially and centrifuged at 400*g* for 10 min at 4°C. The pelleted cells were resuspended in Debris Removal Solution (Miltenyi Biotec) and centrifuged according to manufacturer's instruction. After debris removal, the cells were subsequently resuspended in red blood cell lysis buffer and incubated on ice for 2 min to remove red blood cells. After washing with 1 × PBS, the cell pellets were resuspended in sorting buffer (PBS supplemented with 1% FBS) or cryopreserved until further usage. The cryopreserved cells were thawed and incubated with Dead Cell Removal Kit (Miltenyi Biotec) to remove dead/stressed cells according to manufacturer's instruction.

### 4.3 scRNA‐seq library preparation

scRNA‐seq was performed using 10x Chromium Single Cell 3′ Reagent (V2 Chemistry for Pt1, V3 for all other samples) according to manufacturer's instruction. In brief, viable single cells were resuspended in PBS with 0.04% bovine serum albumin (BSA; Sigma) and counted using the hemocytometer. Cells were then mixed with RT‐PCR master mix and loaded into the Single Cell Chip B and processed through the 10x controller for droplet generation (targeting a recovery of 5000∼8000 cells per sample). After droplet generation, in‐drop lysis and reverse transcription occurs and mRNA transcripts from single cells were uniquely barcoded to identify the cell origin. Following reverse transcription, barcoded cDNAs were purified, amplified by 12 cycles of PCR, end‐repaired, and ligated with Illumina adapters. The resulted libraries were sequenced on the Illumina Novaseq 6000 platform with paired end 150 bp sequencing. Each sample was sequenced to depth of ∼120 G of raw reads.

### 4.4 scATAC‐seq library preparation

scATAC‐seq was performed using 10x Chromium Single Cell ATAC Reagent (V1.1 chemistry) according to manufacturer's instruction. Briefly, viable single cells were resuspended in PBS with 0.04% BSA (Sigma) and counted using the hemocytometer. Cells were subsequently incubated with chilled Lysis Buffer (10 mM Tris‐HCl [pH 7.4], 10 mM NaCl, 3 MgCl2, 0.1% tween‐20, 0.1% IGPEL‐630 [NP‐40 substitute], 0.01% Digitonin, and 1% BSA) for 4 min on ice. Following lysis, the isolated nuclei were combined with ATAC Buffer and ATAC Enzyme (10x Genomics; 2000123/2000138) and then were incubated for 60 min at 37°C. Afterwards, the transposed nuclei were mixed with the Master Mix comprising of Barcoding Reagent (10x Genomics; 2000124), Reducing Agent B (10x Genomics; 2000087) and Barcoding Enzyme (10x Genomics; 2000125/2000139) and subjected to Chromium Chip E (10x Genomics; 2000121). The droplet generation was processed through the 10x controller targeting for 10,000 cells per sample. Resulting single‐cell droplets were collected and linear amplification was performed by PCR with 12 cycles. The sequencing libraries were constructed by amplification of barcoded product with sample index and DNA clean‐up. The libraries were sequenced on Illumina sequencer with the following read length: 50 bp Read 1N, 8 bp i7 Index, 16 bp i5 Index, and 50 bp Read 2N. Each sample was sequenced with depth of ∼20 G of raw reads.

### 4.5 Preprocessing of scRNA‐seq data

scRNA‐seq sequencing data were demultiplexed, aligned, and quantified using the Cell Ranger Single‐Cell Software Suite (version 2.3, 10x Genomics) against the human reference genome hg19 or mouse reference genome mm10. The generated outputs were processed using R package Seurat (version 3.1).^15^ To filter out bad‐quality cells, we considered the following criteria and excluded them: cells with few genes per cell (<50) or many molecules per cell (>20,000); cells for which more than 10% of counts were derived from mitochondrial genes. For samples of Pt7, Pt9, Pt12, Pt14, Pt17, and Pt20, this threshold was set to 30% of mitochondrial genes because these cells were cryopreserved and stressed due to freeze–thaw process. It is worth noting that a more permissive filtering was necessary to avoid filtering out human neutrophils and retain nonhematopoietic cells.^21^ We subsequently combined three methods “DoubletScore,” “scDblFinder,” and “DoubletFinder” to identify and exclude doublet cells. The filtered gene expression matrix was normalized using Seurat's NormalizeData function so that the expression of each gene was multiplied 10,000 and log transformed.

### 4.6 Dimension reduction and unsupervised clustering

To integrate datasets generated from different samples and batches, we used the canonical correlation analysis to integrate them using the top 30 dimensionalities. After the integration, to perform dimension reduction, we first scaled the data by shifting and scaling the expression of each gene so that the mean expression across cells was 0 and the variance across cells was 1. Afterwards we ran PCA analysis using top 2000 genes identified by FindVariableFeatures function of Seurat. We calculated a PCA matrix and chose the first 50 components for *t*‐stochastic neighbor embedding (tSNE) dimension reduction.

### 4.7 Identification of cellular clusters and differentially expressed genes

We used Seurat's FindAllMarkers function for identification of major canonical cellular clusters, by which we identified marker genes and designated cell cluster labels accordingly.

The detailed description of the clusters and corresponding marker genes are included in the main text. For identification of DE genes between clusters, we used Wilcoxon rank sum test, comparing natural log transformed and library size normalized expression values of genes that are expressed in at least 25% of cells between the cluster of interest. The resulted DEGs were filtered using a minimum log2 (fold change) of 0.5 and a maximum FDR value of 0.01.

### 4.8 Construction of single‐cell trajectories

To construct cell development trajectories, we used the Monocle 2 package to align the cells in pseudo‐time order. The DDRTree approach implemented in the reduceDimension function of Monocle 2 was used to map cells.^26^

### 4.9 Assessment of receptor/ligand interactions

To evaluate putative interactions between cells, we used CellPhoneDB and CellTalker.^34^, ^35^ The database of human receptors and ligands (including subunit of heteromeric complexes for both ligands and receptors) was used to identify putative ligand/receptor interactions between cell types. The putative ligands and receptors were determined according to whether they were expressed on each cell. Afterwards, putative interaction pairs were identified and displayed as circos plots.

### 4.10 Tissue enrichment of cell subpopulations

We used observed cell number over the expected cell number (*R*_obs/exp_) of a given cell cluster to determine whether such cluster is depleted or enriched.^8^ The observed and expected cell numbers are obtained from the χ^2^ test. Then the *R*_obs/exp_ was *z*‐score transformed. Therefore, if *R*_obs/exp_ > 0, it means the cell numbers of one cluster are more frequently observed than random expectations, that is, enriched. If *R*_obs/exp_ < 0, it suggests that the cells of one cluster in the specific tissue are depleted.

### 4.11 scATAC‐seq data preprocessing and analysis

Similar to single‐cell RNA‐seq data, scATAC‐seq sequencing data were demultiplexed, aligned, and quantified using the Cell Ranger Single‐Cell Software Suite (version 2.3, 10x Genomics) against the human reference genome hg19. The generated outputs were processed using R package Signac.^15^ We then did quality control assessment and only retained cells that met the following threshold: transcriptional start site (TSS) enrichment score >2; total number of fragments in peaks >1000 and <20,000; fraction of fragments in peaks >20%; the ratio of reads in ENCODE blacklist sites <1.5%.

To reduce dimensions of the scATAC‐seq dataset, we first performed term frequency‐inverse document frequency (TF‐IDF) normalization. The normalized matrix was used as input for TF‐IDF weighting, and term frequency and smoothed inverse document frequency (SVD) were used as weighting scheme. The data were then reduced to 50 dimensions using SVD, and cells were mapped into two dimensions using *t*‐SNE. To find differentially accessible peaks between cell clusters, we used FindMarkers function of Signac R package and performed logistic regression for accessible peaks.

To transfer cell labels from scRNA‐seq clusters to scATAC‐seq data, we quantified the activity of each gene by assessing the chromatin accessibility within 2 kb upstream region that was associated with each gene and created a new gene activity assay using the FeatureMatrix function of Signac. We then identified shared correlation patterns in the gene activity matrix and scRNA‐seq dataset in order to identify matched biological states across the two modalities. Then each cell of scATAC‐seq got the classification score corresponding to individual scRNA‐seq label.

To discover transcription factor dynamics and variation in their motif accessibility, we conducted analysis using chromVAR.^27^ Briefly, we downloaded position weight matrices (PWMs) for 579 known TFs from JASPAR and used FIMO with default parameters to find transcription factor motif occurrences. Transcription factor motif to peak assignments were used in conjunction with counts from 500 bp size fixed cluster‐specific peaks to calculate an accessibility deviation *z*‐score for each transcription factor motif/cell pair.

### 4.12 Whole exome sequencing

Genomic DNA was extracted from either FFPE sections or fresh tissues. The quality of gDNA of each sample was assessed to meet the requirement of purity and concentration (OD260/280 > 1.8; >50 ng/ml). The gDNA was subsequently sonicated to a peak size of ∼250 bp fragments, followed by end‐repairing, dA‐Tailing and adaptor ligation using the NEBNext DNA Library Prep Reagent Set from Illumina (New England Biolabs). The DNA fragments were purified using AMPure beads for desired size selection (300–400 bp). The purified DNA was amplified by PCR with 10 cycles and the resulted PCR products were then subjected to exome sequence capture using SureSelect Human All Exon V6. The enriched elution was amplified by PCR with 10 cycles and the amplicons were size‐checked and quantitated using a BioAnalyzer 2100, and then subjected to 2 × 150 bp paired‐end on the Illumina Novaseq 6000 platform. Each sample was sequenced to 500× coverage depth.

### 4.13 WES data processing and SNV/indel calling

The raw reads were trimmed and filtered for quality, and then aligned to the human genome hg19 using BWA. Reads that did not map, mapped non‐uniquely, mapped to repetitive regions or to chromosome M, as wells as PCR duplicates were removed. The variant calling was performed using the Genome Analysis Toolkit (GATK) and annotated using ANNOVAR. TMB was classified following the below criteria: low ≤20 mutations/Mb, high ≥20.

### 4.14 TCGA data analysis

The TCGA colon adenocarcinoma (COAD) and rectum adenocarcinoma (READ) data were used to confirm the gene expression differences of the T‐cell subtype between patients of hypermutated and nonhypermutated genomes. The gene expression data and genomic mutation data were downloaded from UCSC Xena (http://xena.ucsc.edu/).

### 4.15 Immunohistochemistry staining

Tissues were fixed in 4% paraformaldehyde for 24∼48 h, paraffin embedded, and the specimen were cut in 4‐μm serial sections. Slides were rehydrated through histoclear and series concentration of ethanol, and antigen retrieval was performed in a high‐pressure heat repair process using citrate buffer at pH 6.0. Tissues were blocked in goat serum (ZLI‐9022; ZSGB‐BIO) for 60 min at 37°C, and then incubated with primary antibodies followed by HRP‐linked secondary antibodies and diaminobenzidine (ZLI‐9018; ZSGB‐BIO) staining. Counterstaining was done with hematoxylin for 45 s. Antibodies used are listed as following: CD19 (Cat# GB11061, Servicebio), HLA‐DMB (Cat# bs‐4107R, Bioss), and HRP‐conjugated IgG (Cat#ZB‐2306, Cat# G1215, ZSGB‐BIO).

## CONFLICT OF INTEREST

All of the authors declare no interest of conflicts.

## AUTHOR CONTRIBUTIONS

Guangshuai Jia designed, coordinated, and conceived the project, analyzed the data, and wrote the manuscript. Yan Mei, Weiwei Xiao, Hao Hu, Guanming Lu, Mengdie Lü, Wenhui Ma, and Lingdan Chen prepared samples and performed scRNA‐seq, scATAC‐seq, and WES. Weiwei Xiao, Hao Hu, YuanHong Gao, LiRen Li, Gong Chen, and Zifeng Wang assisted with human sample acquisition. Yan Mei performed immunohistochemistry assay. Lingdan Chen, Zhun Sun, and Ting Jiang provided technical assistance. Pinghong Zhou, Hanjie Li, Duojiao Wu, Zifeng Wang, and Qibin Leng contributed to data processing, discussions and advice, and edited the manuscript.

## Supporting information

Supporting information

Supporting information

Supporting information

Supporting information

Supporting information

Supporting information

Supporting information

Supporting information

Supporting information

Supporting information

## Data Availability

Raw and processed sequencing data are released upon publication. This study did not generate any unique code. All software tools used in this study are freely available. The authors declare that all R scripts supporting the findings of this study are available from the corresponding author upon reasonable request.
