# Supplementary figures and images for: Single‐cell analyses reveal suppressive tumor microenvironment of human colorectal cancer
Source: Clin Transl Med. 2021 Jun 6;11(6):e422. doi: 10.1002/ctm2.422 (PMC8181206; doi:10.1002/ctm2.422)

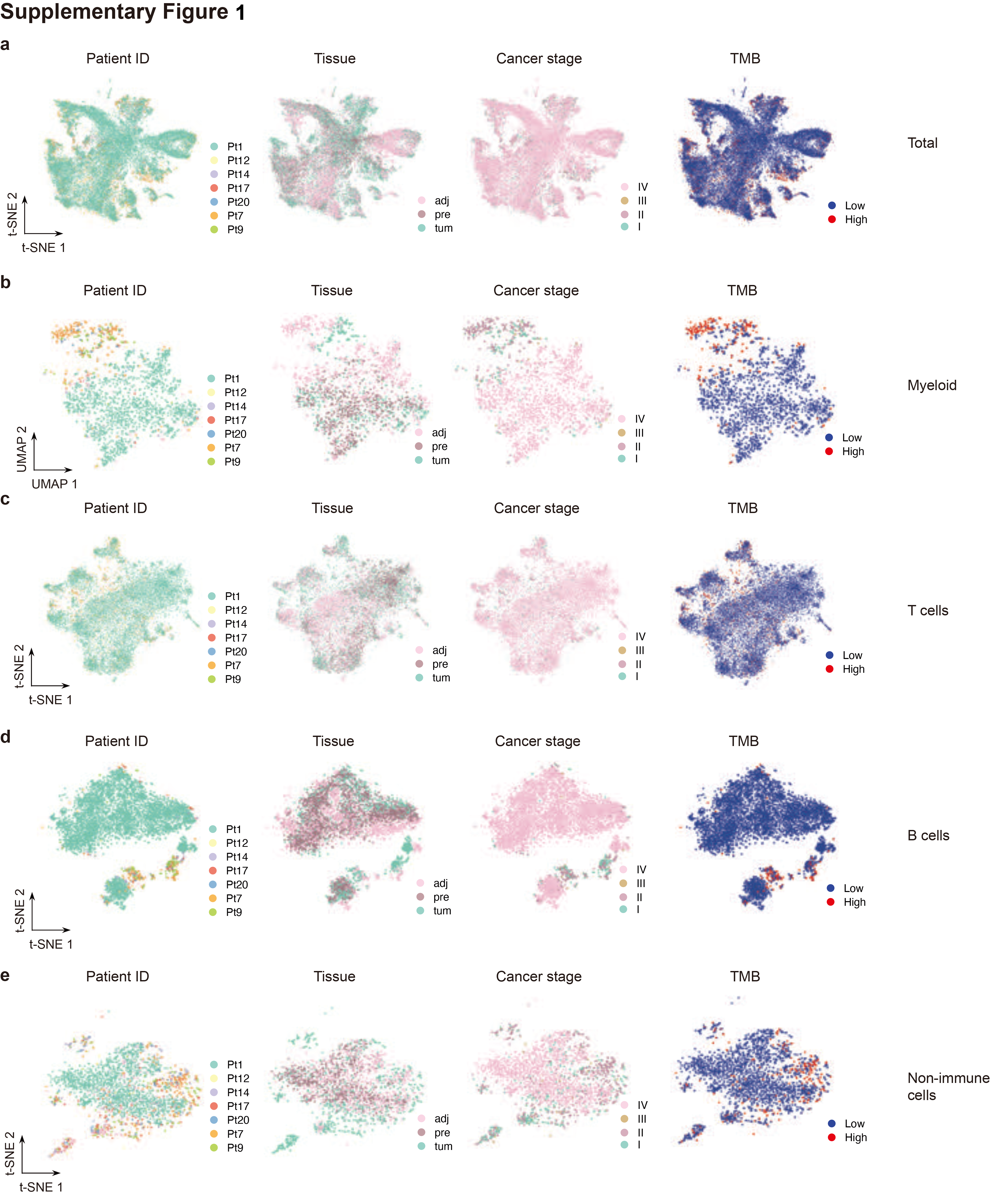

Supplement: Supplementary file 1 — Supporting information [file CTM2-11-e422-s003.tif]

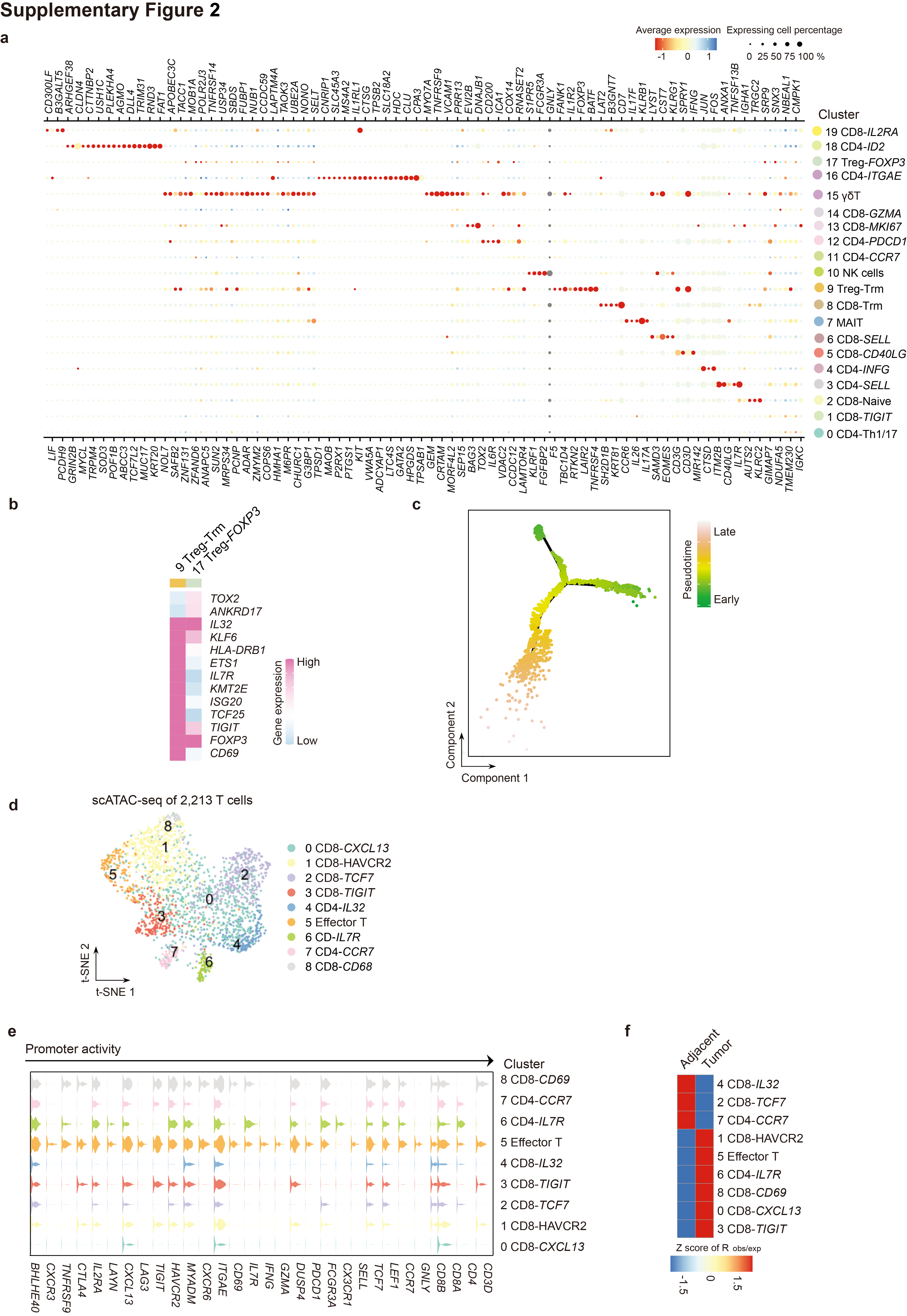

Supplement: Supplementary file 2 — Supporting information [file CTM2-11-e422-s010.tif]

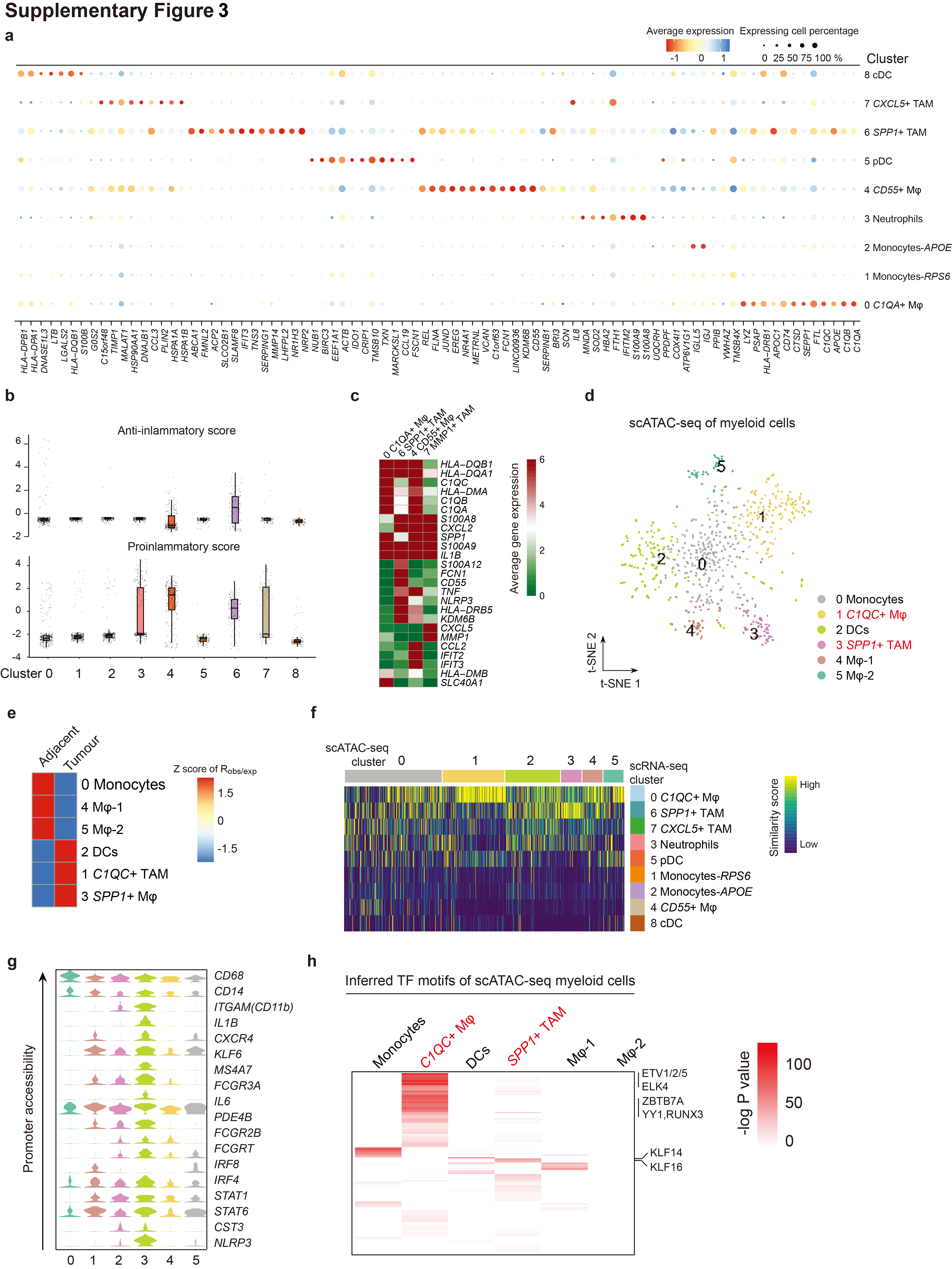

Supplement: Supplementary file 3 — Supporting information [file CTM2-11-e422-s007.tif]

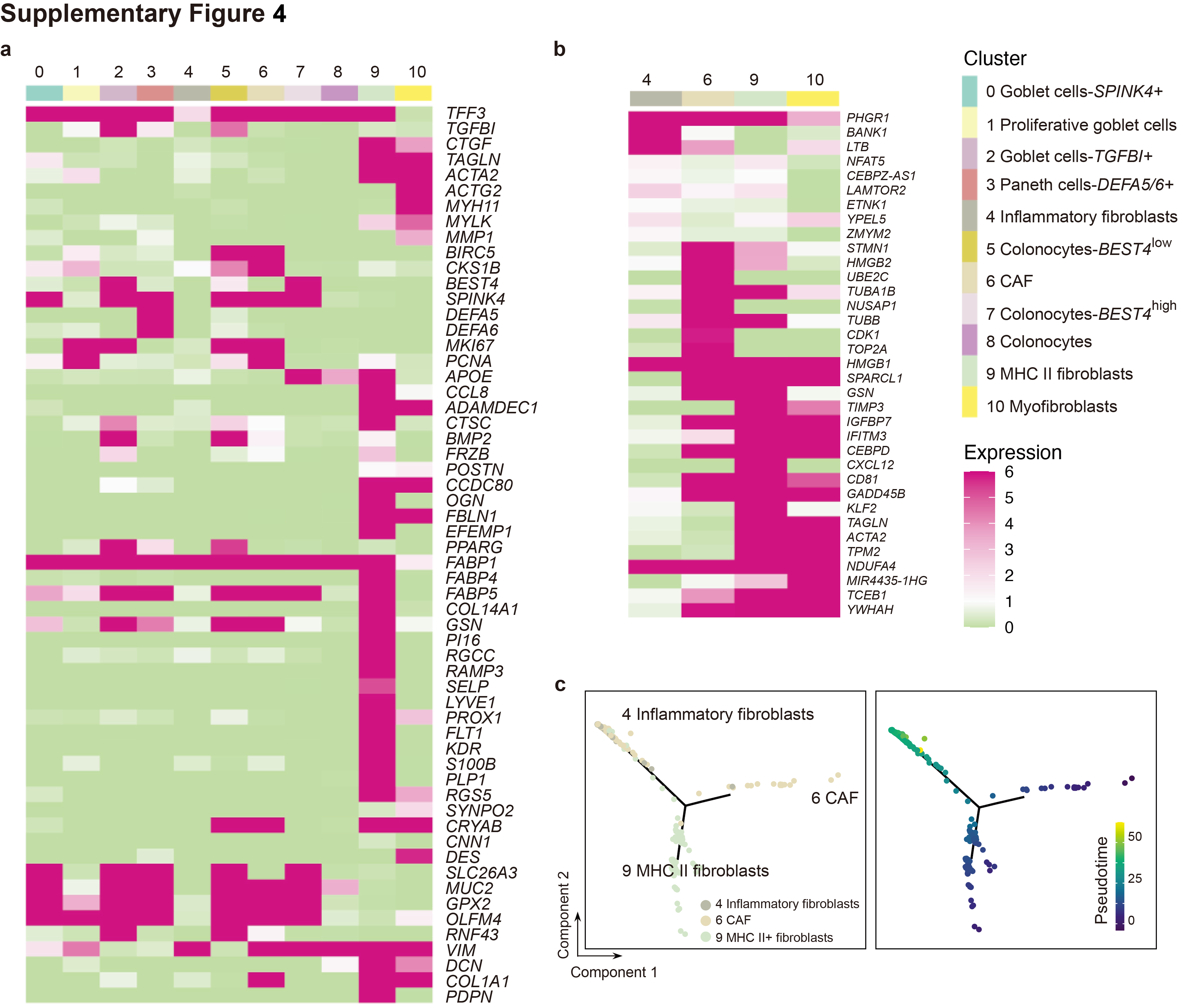

Supplement: Supplementary file 4 — Supporting information [file CTM2-11-e422-s002.tif]

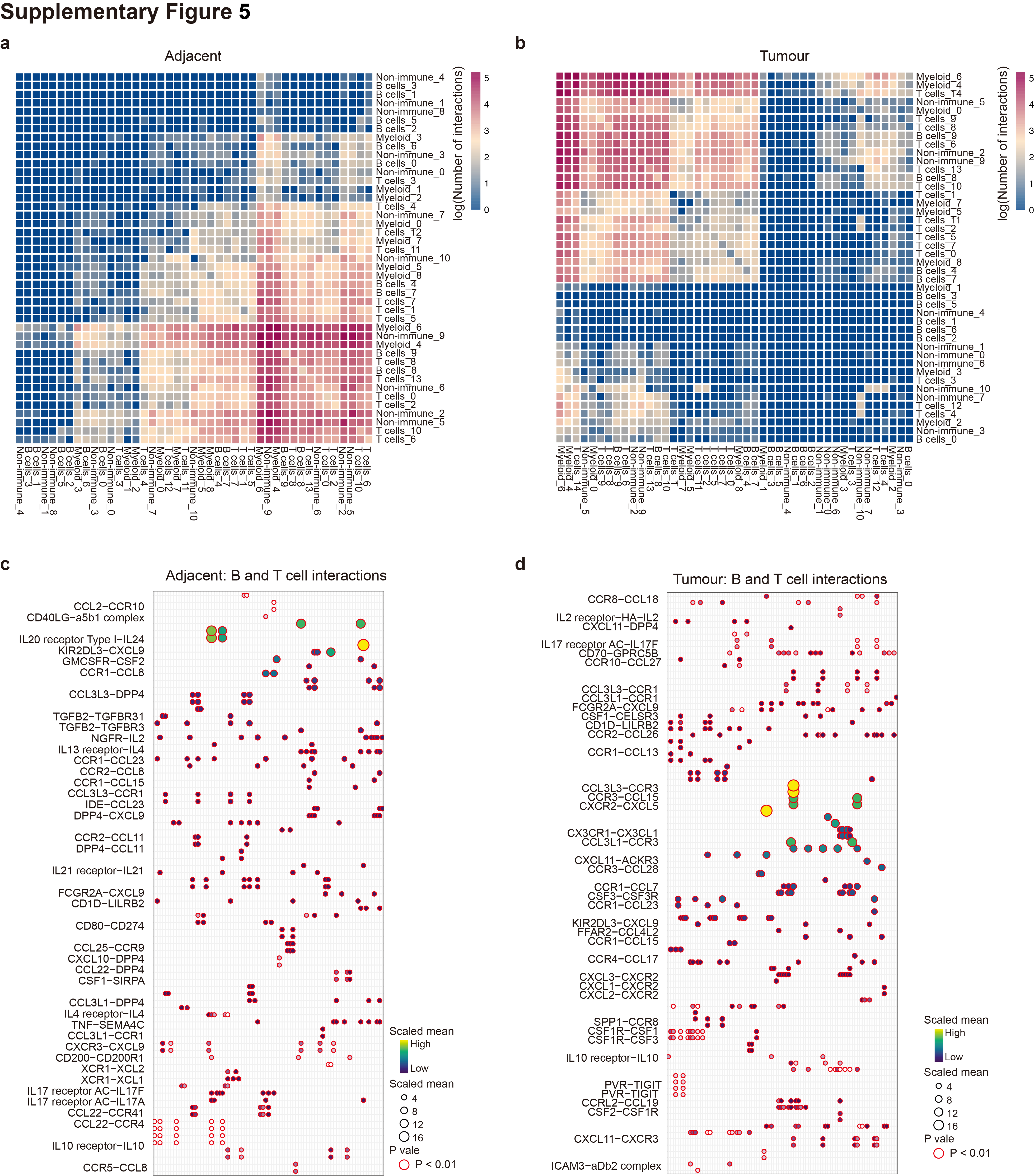

Supplement: Supplementary file 5 — Supporting information [file CTM2-11-e422-s004.tif]
